# Intelligence

**Published:** 1830-02

**Authors:** 


### INTELLIGENCE.

### *Decree of the Court of King's Bench, establishing the Legality of Charges for* Medical Attendance *by General Practitioners.*

The mode by which general practitioners have hitherto been remunerated for their professional labours, by sending medicines to their patients in an expensive form, is no less derogatory from the respectability of the members of a liberal profession, than it is unjust and disgusting to the public. This much to be reprehended custom, however, has not been continued from the *inclination* of the general practitioner; he has been compelled to adopt it to secure to himself a legal demand for the services he had rendered; for, until the present decision by Lord Tenterden, it has been understood that no charge *for medical attendance* could be substantiated by the general practitioner in a court of justice. Every man who desires to raise himself or the body to which he belongs in the estimation of the public, will gladly take advantage of this decree. The general practitioner will henceforth send to his patients such medicines only as may be required, and make a charge for attendance, which must of course be graduated by the circumstances of the party. Many of the leading general practitioners in the metropolis have long indeed acted upon this principle; and, although the appearance of a moderate demand, in a single line, may be objected to by some who would freely pay treble the amount, provided they have the satisfaction of being furnished with a long list of items, under the denomination of pills, powders, and potions, we know from experience that it ultimately tends to the advantage of the practitioner in every point of view. We could indeed mention many instances in which a general practitioner has been particularly selected, by families of the highest respectability, because he had adopted the more honourable plan of sending no medicines that were not absolutely necessary, and of charging for his attendance. The chief difficulty in carrying this plan into general execution is obvious. How much is the general practitioner to charge for each visit? To this question it must be answered, that no definite sum can be abstractedly fixed. Medical attendance must be considered like every other saleable article, and whether it be furnished by the physician or the apothecary, the price of it must fluctuate, according to the means of the consumer. Most physicians are nothing loath to pay several visits for one fee, rather than lose the patient; the general practitioner must of course be guided by the same principle; and, while he endeavours to secure to himself a fair remuneration for the harassing discharge of his duties, from those who are capable of paying for medical attendance as it ought to be paid for, he must never be unwilling to shew the same kindness and attention to those whose circumstances debar them from bestowing an equivalent reward. The following report will be read with no ordinary interest by every general practitioner, all of whom must feel highly indebted to Mr. Handey for the signal service he has rendered to that branch of the profession to which he belongs, by having boldly submitted his very honourable demand to the decision of a court of justice.

COURT OF KING'S BENCH, SATURDAY, JAN. 9, 1830.*

HANDEY *v.* HENSON.

Mr. Thessiger said, that this was an action brought by Mr. James Handey, surgeon and apothecary, against Mr. Henson, of Upper Stamford street, an attorney of this court. The demand was for £7 0 6, for medicines and attendance furnished to the defendant's family. The first attendance was on a child of the defendant, to whom Mr. Handey was first called at night. He continued this attendance for a short time, and the child required but little medicine. He thought it proper to state here to the jury, that Mr. Handey had adopted a line of conduct in his medical practice, which he (Mr. T.) considered highly honourable and respectable. It was that of not sending in large quantities of useless medicines, but attending when necessary, and charging for his professional talents and visits. Having mentioned this circumstance, he referred to the next occasion, on which a charge was made by the plaintiff. The case was that of Mrs. Henson, the defendant's wife, who had a severe eruption of the face, which the plaintiff told her might be re-

* Lancet.

lieved with little medicine, which was accordingly furnished. After an attendance of a little more than five weeks, the plaintiff charged five guineas for the medicines and visits, in one charge. This formed the second part of the bill; the third, and last, was for attendance on the defendant's mother-in-law, in which the same honourable mode of procedure was observed. The only difficulty which presented itself to him (Mr. Thessiger) was, that of proving the visits; but he trusted he should show to the entire satisfaction of the jury, by the evidence he meant to produce, that the medicines were supplied, and the attendances furnished, for which the charges were made. He should now call his witnesses.

Lord Tenterden. Is the plaintiff qualified under the act, or was he in business before the year 1815.

Mr. Thessiger. He was in business before 1815, my Lord. I will call Mr. White.

Mr. James White, of the firm of White and Cautherly, proved having supplied the plaintiff with drugs, and having waited on him three times prior to the year 1815, and that the plaintiff was in practice as a general practitioner before that time.

Mr. Jacob Dixon sworn: Was an apprentice of the plaintiff at the time of these visits; recollects the plaintiff being sent for in the night to the child, and his subsequent putting up and sending out the medicines necessary for Mrs. Henson's disorder. Had applied from fifteen to twenty times for the amount; judged what was the nature and state of the disorder from what the plaintiff communicated to him, and saying what the medicines were for. Defendant's wife had frequently promised to pay the bill. In answer to a question from Lord Tenterden, the witness said he could never see the defendant, either at his own house or his office. The defendant seemed to avoid him.

Mr. James Thorn was called to prove the remainder of the bill. Recollected not only putting up, but frequently taking the medicines to the house himself.

Mr. William Lobb, surgeon, of Aldersgate street, had seen the plaintiff's bill, and considered tbe charges perfectly fair and reasonable.

Mr. Platt, for the desendant, commented generally on the plaintiff's case. He admitted the first and latter parts, and objected only to the charge in the bill of £5 5 0. He called upon the jury to look at the account, in which the charges for medicines amounted to about 50s., leaving the remainder to be made up by visits, which there was no proof had ever been made; this, he thought, entitled him to their verdict. Mr. Platt called no witness.

Lord Tenterden. Gentlemen of the jury, this action has been brought, as you have heard stated, by Mr. Handey, a respectable surgeon, residing in Waterloo Bridge road, against the defendant, Mr. W. S. Henson, an attorney of this court, for the recovery of the sum of £7 0 6, for medicines and attendance. The first and last items are not disputed. In one part of the bill there is a charge of five guineas, which appears to be for five weeks' attendance and medicines. There does not seem to be much dispute as to the charge for the medicines, but for the visits; and of these, it is said, there is no proof; but I cannot see how a medical man is to prove these attendances. It may be said that when he makes them, he has his servant behind his carriage, or with him; but what can that servant prove? The opposing counsel says, that the persons in the house of the patient might be called to prove the attendances, but how are these servants or persons to be got at, or how are their names to be obtained? I think, Gentlemen, that the plaintiff has proved as much as can be expected; and Mr. Dixon proves that, on the plaintiff's return from his daily professional calls, the visits were entered in a book by him under the plaintiff's order, or by Mr. Handey himself. I cannot see, if a medical gentleman pursues the same honourable plan which this gentleman has done, of not sending in large and useless quantities of medicine, how he is to be remunerated, but by being paid for his attendances. I will hand you the bill, which you will please to inspect; and, from the evidence given, you will say whether you consider this to be a fair and just demand or not, and give a verdict accordingly.

The jury, after a few minutes' consideration, returned a verdict for the plaintiff, damages £7 0 6, and costs.

The following is a copy of that part of the bill which was objected to by the defendant:

| | | |
|---|---|---|
| July 19. | The mixture | |
| | The pills | |
| | The lotion | |
| 20. | The pills. | |
| | The two draughts | |
| 21. | Visit | |
| 22. | Visit | |
| 22. | The box of pills | |
| | The lotion | |
| 23. | Visit | |
| 24. | Visit | |
| | The draught | |
| 25. | Visit | |
| 26. | The box of pills | |
| | The draught | |
| 27. | Visit | |
| 28. | Visit | |
| 28. | The lotion | |
| 30. | The box of pills | £5 5 0. |
| 31. | Visit | |
| Aug. 2. | The draughts | |
| | The box of pills | |
| 4. | The draughts | |
| 5. | Visit | |
| 6. | Visit | |
| 8. | The box of pills | |
| | The draughts | |
| 10. | Visit | |
| | The lotion | |
| 12. | The box of pills | |
| | The draughts | |
| 15. | Visit | |
| 16. | Visit | |
| 17. | Visit | |
| Sept. 5. | Visit | |
| 16. | The lotion | |

---

The Medical Society of Louvain have offered a prize of a gold medal for the author of the best Treatise on the General and Comparative Diagnosis of Acute and Chronic Diseases of the Cerebro-spinal System and its Membranes.

### BILLS OF MORTALITY.

### DISEASES.

| | | | | | |
|---|---|---|---|---|---|
| Abscess | 124 | Erysipelas | 42 | Palpitation of the Heart | 7 |
| Age and Debility | 2076 | Fever | 1167 | Palsy | 18 |
| Apoplexy | 429 | ——, Intermittent, or Ague | 53 | Paralytic | 185 |
| Asthma | 1131 | ——, Typhous | 103 | Pleurisy | 21 |
| Bedridden | 2 | Fistula | 7 | Rheumatism | 45 |
| Bile | 11 | Flux | 4 | Scrofula | 6 |
| Cancer | 94 | Grief | 5 | Small-pox | 736 |
| Childbirth | 264 | Gout | 33 | Sore Throat, or Quinsey | 28 |
| Consumption | 5251 | Hemorrhage | 38 | Spasm | 51 |
| Contraction of the Heart | 9 | Hernia | 26 | Stillborn | 933 |
| Convulsions | 2761 | Hooping Cough | 633 | Stone | 19 |
| Croup | 123 | Hydrophobia | 4 | Stoppage in the Stomach | 24 |
| Diabetes | 3 | Inflammation | 2385 | Stricture | 4 |
| Diarrhœa | 31 | Inflammation of the Liver | 197 | Suddenly | 126 |
| Dropsy | 1021 | Insanity | 258 | Teething | 541 |
| —— on the Brain | 855 | Jaundice | 32 | Thrush | 82 |
| —— on the Chest | 106 | Jaw-locked | 2 | Tumor | 16 |
| Dysentery | 6 | Measles | 578 | Venereal | 11 |
| Enlargement of the Heart | 40 | Miscarriage | 8 | Worms | 7 |
| Epilepsy | 67 | Mortification | 286 | | |
| Eruptive Diseases | 28 | Ossification of the Heart | 16 | Total of Diseases | 23,169 |

### CASUALTIES.

| | | | | | |
|---|---|---|---|---|---|
| Broken Limbs | 2 | Fractured | 1 | Overlaid | 2 |
| Broken Ribs | 1 | Frighted | 1 | Poisoned | 7 |
| Burnt | 53 | Frozen | 2 | Run over | 4 |
| Choked | 1 | Killed by Falls and other Accidents | 75 | Scalded | 2 |
| Drowned | 136 | Killed by Fighting | 1 | Strangled | 1 |
| Excessive Drinking | 3 | Killed themselves | 35 | Suffocated | 10 |
| Executed* | 8 | Murdered | 4 | | |
| Found dead | 6 | | | Total of Casualties | 355 |

Christened { Males.... 13,674; Females 13,354 } In all, 27,028 | Buried { Males.. 12,015; Females 11,509 } In all, 23,524

Whereof have died,

| | | | | | |
|---|---|---|---|---|---|
| Under two years of age | 6710 | Thirty and forty | 1902 | Eighty and ninety | 749 |
| Between two and five | 2347 | Forty and fifty | 2092 | Ninety and one hundred | 95 |
| Five and ten | 1019 | Fifty and sixty | 2094 | One hundred and one | 1 |
| Ten and twenty | 949 | Sixty and seventy | 2158 | One hundred and eight | 2 |
| Twenty and thirty | 1563 | Seventy and eighty | 1843 | | |

Increased in the Burials reported this year, 1,815.

* There have been executed within the Bills of Mortality, 26; of which number, only 8 have been reported as such.

### METEOROLOGICAL JOURNAL,

By Messrs. Harris and Co. Mathematical Instrument Makers, 50, High Holborn.

| December | Moon | Rain gauge. | Thermom. | | | Barometer. | | De Luc's Hygrom. | | Winds. | | Atmospheric Variations. | | |
|---|---|---|---|---|---|---|---|---|---|---|---|---|---|---|
| | | | 9 a.m. | max. | min. | 9 A.M. | 10P.M. | 9 A.M. | 10P.M. | 9 A.M. | 10P.M. | 9 a.m. | 2 p.m. | 10 p.m. |
| 20 | | | 33 | 36 | 27 | 29.73 | 29.77 | 67 | 67 | NE | NE | Foggy | Fine | Fine |
| 21 | | | 30 | 33 | 28 | .80 | .70 | 67 | 70 | NW | W | —— | Snow | Fine |
| 22 | | | 33 | 34 | 24 | .69 | .62 | 70 | 65 | ENE | E | F.& Sn. | Cloudy | Fine |
| 23 | | | 30 | 33 | 25 | .62 | .55 | 65 | 66 | NE | NE | Foggy | Cloudy | Foggy |
| 24 | | | 27 | 32 | 22 | .61 | .73 | 67 | 67 | NE | NE | Snow | Fine | Fine |
| 25 | | | 24 | 30 | 25 | .86 | 30.00 | 66 | 64 | NE | NE | Fine | Snow | Snow |
| 26 | ● | | 26 | 26 | 24 | 30.21 | .22 | 64 | 64 | ENE | NNE | Fine | Fine | Cloudy |
| 27 | | | 26 | 28 | 19 | .21 | .20 | 65 | 65 | NE | NE | Foggy | Foggy | Fine |
| 28 | | | 22 | 28 | 21 | .20 | .15 | 65 | 65 | N | NNE | —— | Cloudy | Snow |
| 29 | | | 24 | 27 | 24 | .23 | [illegible] | 65 | 67 | SE | NE | —— | —— | Snow |
| 30 | | | 27 | 31 | 27 | .31 | .31 | 67 | 67 | ENE | NE | —— | Fine | Fine |
| 31 | | | 31 | 32 | 29 | .40 | .44 | 66 | 66 | E | E | —— | Cloudy | Cloudy |
| Jan. | | | | | | | | | | | | | | |
| 1 | | | 32 | 34 | 30 | .50 | .47 | 66 | 67 | NE | NE | —— | Foggy | Cloudy |
| 2 | ☽ | | 32 | 34 | 32 | .38 | .37 | 67 | 67 | N | ENE | —— | Fine | Cloudy |
| 3 | | | 34 | 35 | 33 | .29 | .18 | 69 | 69 | ENE | NE | Rain | Cloudy | Cloudy |
| 4 | | | 36 | 37 | 35 | .14 | .14 | 70 | 72 | NNE | NE | Rain | Sleet | Sleet |
| 5 | | | 37 | 39 | 30 | .12 | .18 | 72 | 72 | N | NNW | Foggy | Foggy | Fine |
| 6 | | | 32 | 34 | 32 | .19 | .02 | 72 | 73 | NNW | ENE | —— | Fine | —— |
| 7 | | | 38 | 42 | 33 | 29.93 | 29.80 | 73 | 69 | W | N | —— | Show'ry | Cloudy |
| 8 | | | 35 | 38 | 32 | .94 | 30.01 | 67 | 68 | N | N | Show'ry | Cloudy | Fine |
| 9 | ○ | | 34 | 39 | 30 | 30.05 | 29.83 | 70 | 70 | N | NNW | Foggy | Fine | Cloudy |
| 10 | | | 33 | 39 | 32 | 29.72 | .65 | 68 | 63 | NW | NW | Fine | —— | Fine |
| 11 | | | 35 | 35 | 32 | .57 | .51 | 60 | 65 | NNW | N | —— | Snow | Snow |
| 12 | | | 32 | 33 | 30 | .70 | .84 | 65 | 67 | NNE | NNE | Snow | Cloudy | Cloudy |
| 13 | | | 32 | 32 | 26 | .77 | .65 | 67 | 67 | NNE | ESE | Foggy | Snow | Cloudy |
| 14 | | | 28 | 32 | 28 | .78 | .81 | 65 | 67 | ENE | E | —— | Cloudy | Cloudy |
| 15 | | | 32 | 34 | 27 | .79 | .76 | 67 | 67 | E | E | —— | —— | Sleet |
| 16 | | | 29 | 32 | 22 | .79 | .82 | 67 | 67 | E | SE | —— | —— | Fine |
| 17 | ☾ | | 22 | 25 | 19 | .82 | .84 | 66 | 65 | NE | NE | Fine | Fine | —— |
| 18 | | | 19 | 23 | 17 | .85 | .70 | 65 | 66 | ESE | SSW | Foggy | Foggy | —— |
| 19 | | | 20 | 31 | 20 | .55 | .40 | 66 | 69 | SSW | SE | —— | Fine | Cloudy |

In consequence of the late frost, the Rain cannot be ascertained.

